# Phenology of the elongate hemlock scale (Hemiptera: Diaspididae) on Fraser fir Christmas trees in western North Carolina

**DOI:** 10.1093/jee/toag098

**Published:** 2026-04-23

**Authors:** Dominic Manz, Kelly L F Oten, Clyde E Sorenson, Justin G A Whitehill, Robert M Jetton

**Affiliations:** Department of Forestry and Environmental Resources, North Carolina State University, Raleigh, NC, USA; Department of Forestry and Environmental Resources, North Carolina State University, Raleigh, NC, USA; Department of Entomology and Plant Pathology, North Carolina State University, Raleigh, NC, USA; Department of Forestry and Environmental Resources, North Carolina State University, Raleigh, NC, USA; Department of Forestry and Environmental Resources, North Carolina State University, Raleigh, NC, USA

**Keywords:** forest health, invasive species, armored scale

## Abstract

Understanding the seasonal phenology of an insect pest in a specific region on a specific host is fundamental to the timing of management actions. The elongate hemlock scale, *Fiorinia externa* Ferris (Hemiptera: Diaspididae), is an invasive insect from Japan known to infest various conifer hosts in its invasive range in eastern North America. The phenology of the scale has been studied on hemlock (*Tsuga* spp.) hosts in its native range and portions of its invasive range in the northeastern and mid-Atlantic regions of the United States; similar studies are lacking for the southeastern region. In the Southern Appalachians, this scale poses a significant management and regulatory challenge for Fraser fir (*Abies fraseri* [Pursh] Poir.,) Christmas tree production. The objective of this study was to examine the seasonal phenology of the scale in the western North Carolina production region. Biweekly samples were collected from Fraser fir at three sites over 2 years and analyzed for abundance of each life stage. We found all life stages present at all locations throughout the year. Large variability in egg abundance was observed across 2 years. There was little variability in the abundance of life stages between sampling locations. Substantially more scale eggs, crawlers, 2nd instar nymphs and adult females were observed on the 2 most recent years’ needles as opposed to older needles. These findings can help optimize the timing of management practices to control the elongate hemlock scale more effectively.

## Introduction

The elongate hemlock scale, *Fiorinia externa* Ferris (Hemiptera: Diaspididae), is an invasive insect in North America that is native to eastern Asia where it feeds primarily on hemlocks (*Tsuga* spp.). The scale has a broad host range in North America that includes true firs (*Abies* spp.), cedars (*Cedrus* spp.), spruces (*Picea* spp.), pines (*Pinus* spp.), and yews (*Taxus* spp.) (Dale et al. 2019, Darr et al. 2022, Sidebottom and Bookwalter 2025). First detected in Queens, NY in 1908, it has subsequently spread across the eastern United States from Maine south to Georgia and west into Ohio, Michigan, and Tennessee (Sidebottom and Bookwalter 2025, Fig. 1A).

**Fig. 1.**
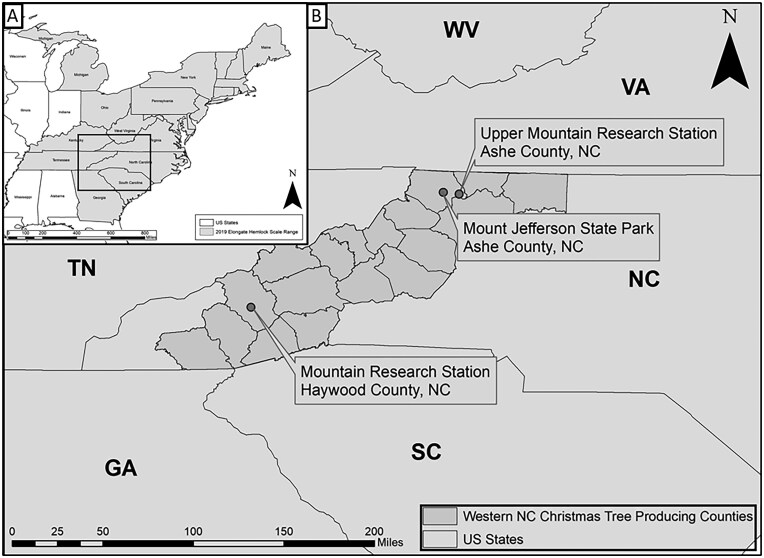
A) Map of Elongate Hemlock Scale range expansion by 2019. Source: USDA Forest Service, Northern Research Station and Forest Health Protection. “Alien Forest Pest Explorer–species map,” https://www.nrs.fs.fed.us/tools/afpe/maps/, and B) Map of western North Carolina Christmas tree producing counties, indicating three collection sites for the phenology study. Locations are displayed with red dots and labelled with name and location. Source: ArcGIS, License: North Carolina State University, Creator: Dominic Manz.

Fraser fir (*Abies fraseri* [Pursh] Poir.) Christmas trees are one of North Carolina’s most important specialty crops, with 850 growers producing ∼50 million trees across 39,000 acres (North Carolina Christmas Tree Association. n.d.a., National Agricultural Statistics Service 2019). Fraser fir makes up >95% of Christmas trees grown in North Carolina. Each year, 5–6 million trees are harvested with an annual wholesale and retail value of $100 million and $250 million, respectively (Owen 2016). Value-added products such as greenery for wreaths and other holiday decorations generate an additional $25 million. Most years North Carolina ranks second in the nation in number of Christmas trees harvested. Trees are shipped to all 50 U.S. states, Canada, Mexico, Japan, Bermuda, the Caribbean Islands, and other international destinations (North Carolina Christmas Tree Association. n.d.b).

Fraser fir production in North Carolina is plagued by an array of diseases and arthropod pests. Recent reports note 15 arthropods and 10 pathogens of concern (Sidebottom 2021, Darr et al. 2022), requiring management costing growers ∼$60/ac/yr, not including equipment costs (Nzokou and Leefers 2007). The effects of these pests range in severity from those that cause significant tree decline and mortality, to those that cause aesthetic damage and reduce tree marketability, to those that become a nuisance post-harvest by infesting peoples’ homes or are transported to new areas on trees shipped long distances. Well-developed integrated pest management programs for many of these pests exist that when properly implemented mitigate their potential impacts (Darr et al. 2022).

Among the significant arthropod pests of Fraser fir, the elongate hemlock scale, has proven to be one of the most challenging to manage. The elongate hemlock scale was first detected on Fraser fir Christmas trees in North Carolina in 1993 (Sidebottom and Bookwalter 2025). Rarely a problem during the 1990s, the insect has increasingly become an issue since 2000 (Sidebottom and Bookwalter 2025) owing to management difficulties associated with its hard covering which limits pesticide efficacy, and its complex life cycle with multiple, overlapping generations affecting application timing. By 2013, 53% of growers reported infestations in their fields (Dale et al. 2019). While the scale has been documented to cause decline like symptoms (needle yellowing, needle cast, branch dieback, stunted growth, occasional tree death) on *Tsuga* spp. native to the eastern United States (Venette et al. 2024), it is not known to cause substantial damage to Fraser fir and it is considered an important aesthetic and regulatory pest on this host (Dale et al. 2019, 2020, Darr et al. 2022). Needle yellowing caused by feeding and the presence of the scale itself both reduce the appearance and marketability of trees, while the post-harvest movement of trees is a potential pathway for the insect being introduced to new areas and hosts (Dale et al. 2020, Darr et al. 2022). Management options for this insect include several chemical controls which, when properly timed, limit adverse impacts on natural enemy populations that also contribute to scale control, as well as cultural controls such as reduced nitrogen fertilization and avoiding interplanting (un-even aged management). Together these strategies can reduce elongate hemlock scale infestations to mitigate aesthetic problems, but complete control cannot be guaranteed meaning scale populations can recover quickly from residual populations (McClure 1977a, Darr et al. 2022).

Throughout its native and invasive range the elongate hemlock scale follows the same life cycle. Female scales complete three life stages after the egg, 1st instar (the mobile phase also called crawlers), 2nd instar and adult; the latter 2 stages are sessile and protected by their covering (McClure 1978, 1979, 1986, Raupp et al. 2008). The male goes through 2 additional stages after the 2nd instar, the prepupal and the pupal stage (henceforth referred to as 3rd/4th instar males), before maturing to adulthood (McClure 1979, Raupp et al. 2008, Dale et al. 2019). Adult males do not feed and die within a few days or after mating (Lambdin et al. 2005, Cowles 2011).

On its native hosts, northern and southern Japanese hemlock (*Tsuga diversifolia* and *T. sieboldii*), elongate hemlock scale completes 2 distinct annual generations (McClure 1986). The first- and second-generation crawlers are present between May and June and July and August, respectively (McClure 1986). Eastern hemlock (*Tsuga canadensis*) is the preferred host in the northeastern U.S., on which the scale completes one and possibly a partial second overlapping generation annually (McClure 1978, 1979, Abell and Van Driesche 2012). Peak crawler emergence of the first generation is between June and July (McClure 1978). The second partial generation crawlers hatch between late September and October and overwinter mainly as gravid females (McClure 1978). In the mid-Atlantic region of the US, the scale completes 2 overlapping generations annually (Stimmel 1980, Abell and Van Driesche 2012). Peak crawler emergence is between late May and early June (Sidebottom and Bookwalter 2025).

Our current understanding of elongate hemlock scale phenology comes from its native range as well as the northeastern and mid-Atlantic region of the U.S. However, this research was done exclusively with hemlocks (*Tsuga* spp.) as the host. Thus, we lack data on the phenology of elongate hemlock scale on Fraser fir in the southern Appalachians. Because the phenology of an insect is expected to vary between hosts and geographic locations (McClure 1978, 1983, 1986, Abell and Van Driesche 2012), the accuracy of predictions of pest population dynamics outside the studied area and host is limited, ultimately affecting management success. It is thus important to study the phenology of this scale on Fraser fir in the southern Appalachians to make informed management decisions. The objective of this project was to map the presence and abundance of all the elongate hemlock scale life stages across three locations and 2 years in western North Carolina to guide management activities of the insect on Fraser fir Christmas trees.

## Methods

### Study Area

This study was conducted on three sites in western North Carolina (Fig. 1B), including 2 sites in Ashe County in northwestern North Carolina (the Upper Mountain Research Station in Laurel Springs and Mount Jefferson State Park in Jefferson). The third location was in Haywood County at the Mountain Research Station in Waynesville in the southwestern region of North Carolina.

### Site Descriptions

The Upper Mountain Research Station Fraser fir clone bank (“UMRS,” 36.393296, −81.308408, Fig. 1B) is 2 ha in size and contains over 650 Fraser fir. The site is steep and situated around a trough with both north and south facing slopes. The elevation of all fields is 850 m. According to the US Climate Data website, annual temperatures range between −7 °C and 27 °C and mean monthly rates range between 97 mm and 131 mm for precipitation and 0 mm and 178 mm for snowfall (US Climate Data). The trees are 15 years old and between 3 m and 6 m tall. Before and during the study, chemical management included application of dinotefuran via high pressure sprayer in April 2020, esfenvalerate and dimethoate via high pressure sprayer in March 2021 and bifenthrin to the foliage of trees using an orchard sprayer in April 2022 (Tracy Taylor, personal communication).

The Mount Jefferson State Park site (“SP,” 36.407511, −81.435948, Fig. 1B) is in Jefferson, North Carolina. The field is divided into three sections with a total acreage of 3 ha and contains over 850 trees. The trees are roughly 20 years old and between 4.5 m and 7.5 m tall. The annual low and high temperatures are −7 °C and 27 °C, respectively. Monthly average precipitation range between 83 mm and 124 mm, according to the US Climate Data (US Climate Data).

The third collection site is at the Mountain Research Station (“MRS,” 35.489680, −82.971621, Fig. 1B) in Waynesville, North Carolina. The field is 1.2 ha and contains over 600 trees. The trees are 15 years old and between 3 m and 6 m tall. Annual temperatures range between −6 °C and 28 °C. Mean monthly precipitation rates vary between 67 mm and 114 mm while mean monthly rates of snowfall range between 0 mm and 102 mm, according to the US Climate Data (US Climate Data). The trees on this site are part of the USDA and North Carolina State University clone bank. Trees have not been treated with insecticides for 4+ years (Justin Whitehill/William Whittier, personal communication, 9 November 2022).

### Sampling Procedure

Branches infested with elongate hemlock scale were collected every 2 weeks or 49 sampling periods between May 2021 and May 2023. During each collection, infested branches were taken from five randomly selected trees in each of the three sites. Initial assessment for infestation was conducted using a hand lens and observations of fluorescent yellow spots to confirm the presence of live scale. If live elongate hemlock scale were observed, up to 2 branches per tree including three needle age classes (new growth, one year old growth, 2 year old growth) between 10 and 68 cm long were removed for analysis, bagged in self-sealing bags, and placed on ice in a cooler, then transported to the NC State University Forest Health Lab in Raleigh, and examined microscopically.

### Data Collection

For each sampling period, we collected between 100 and 125 live scales per tree, for the majority of samples, with some outside of this range. To confirm an insect was alive, in the absence of fluorescent yellow color, the scale’s protective covering was punctured and observed for active bleeding (Dale et al. 2020). The number of crawlers, 2nd instars, 3rd/4th instar males (prepupa and pupal stage) as well as adult females and the number of eggs were recorded. We also counted scale exuviae for each needle and recorded if scales were parasitized by insects or infected with fungi. All observations were recorded for three needle age classes where 0 = needles produced the year of sampling, 1 = needles produced the year prior and 2 = needles produced 2 years prior. An even number of needles was sampled for each needle age class.

### Statistical Analysis

A mixed model analysis of variance was performed using the PROC MIXED procedure in SAS version 9.4 (SAS 2020) to test the main effects of sampling period and sampling year with random variables of sampling site and the abundance of elongate hemlock scale life stages (eggs, crawlers, 2nd instars, 3rd/4th instar males, and adult females). ANOVA was also performed to test the main effect of needle age class on the abundance of elongate hemlock scale life stages. Where statistical differences were found mean separation analysis was performed using Tukey-Kramer HSD. Prior to analysis, data was tested for homogeneity of variance using the PROC Univariate procedure in SAS 9.4 (SAS 2020), and the data were found to be normally distributed. Statistical significance was based on alpha level 0.05.

## Results

**Fig. 2.**
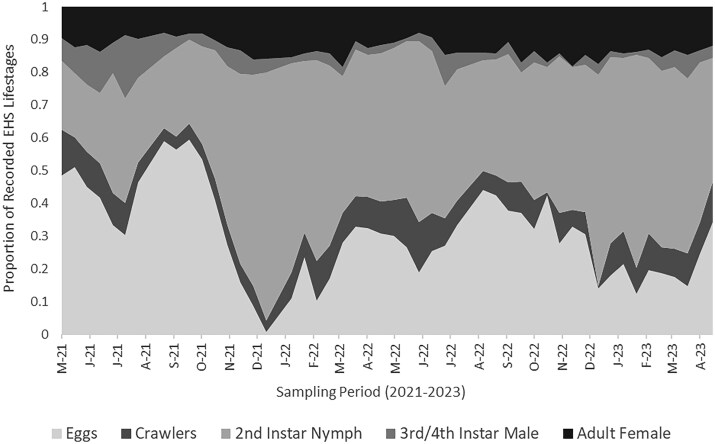
Average proportions of EHS life stages recorded across three locations in western North Carolina (UMRS, Mount Jefferson SP, MRS), over 2 years between May 2021 and May 2023.

Between 24 May 2021 and 8 May 2023, 77,851 live elongate hemlock scale were observed. All life stages were present throughout the study period. The most abundant life stages were 2nd instars and followed by eggs with mean proportions of 44.0% and 30.3%, respectively (Fig. 2). The least abundant stages were 3rd/4th instar males, crawlers and adult females with mean proportions of 4.5%, 8.3%, and 12.8%, respectively (Fig. 2). We observed an average parasitization of 2.15% by suspected parasitoid wasps and infection of 1.14% by suspected entomopathogenic fungi documented in the area (Lovett et al. 2024). On the 15 trees randomly sampled per collection period, the parasitization by insects ranged from 0% to 6.66% and fungal infection from 0% to 5.08%. Across the 2-year study period and all locations, we found an average (±SE) of 6.7 ± 0.1 eggs per gravid female, with a minimum, median and maximum of 2.0, 6.5, and 17.0 eggs, respectively.

**Fig. 3.**
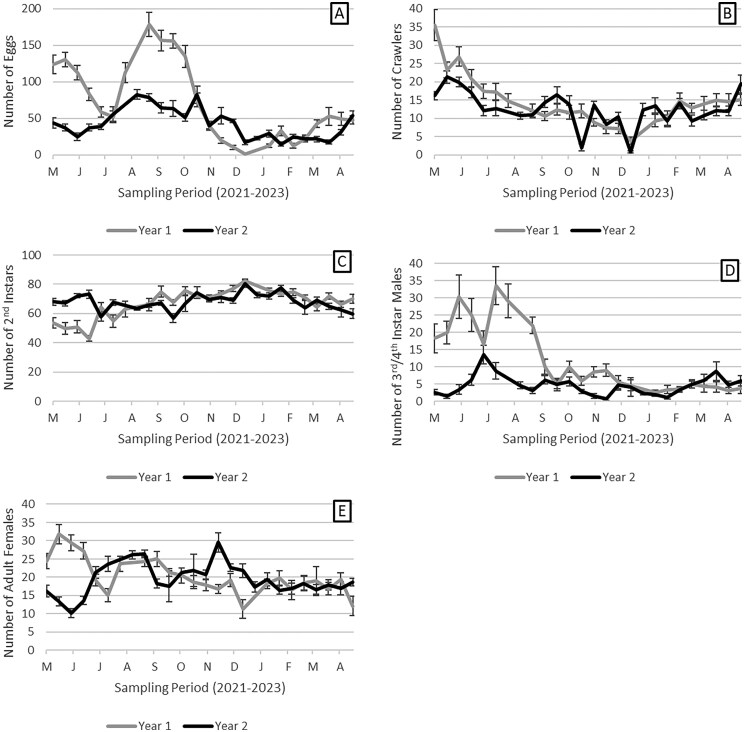
Average distribution of life stage abundance per tree per collection period and year. The data are averaged across the three locations (239 trees at the UMRS, 233 trees at Mount Jefferson SP and 235 trees at the MRS) over the 2-year study period. The distribution of abundance of the A) egg, B) crawler, C) 2nd instar, D) 3rd/4th instar male, and E) adult female stages.

Abundance of all life stages was significantly affected by sampling period and the interaction between sampling year and sampling period (Table 1). Further, abundance of the egg, crawler and 3rd/4th instar male stages were significantly affected by sampling year and all abundance of all stages, except the egg stage, were significantly affected by sampling site (Table 1). The abundance of crawlers, for Years 1 and 2, is highest between early May and mid-July and lowest between mid-November and early January (Fig. 3B). The abundance of 2nd instar is highest in early January and lowest between mid-April and mid-July (Fig. 3C). The abundance of 3rd/4th instar male stage is highest between late May and mid-September in Year 1 as well as mid-April and Mid-July in Year 2. Lowest abundance for Years 1 and 2 were between mid-December and early May and early November and late February, respectively (Fig. 3D). Numbers of adult females observed were highest between late May and early July of Year 1 and between mid-July and mid-September of Year 2, with an additional peak in early December. Large variation in adult female abundance was observed between late May and early January of sampling Years 1 and 2 (Fig. 3E).

**Table 1.** Statistical outputs from the Linear Mixed Model (top) and the General Linear Model (ANOVA) (bottom) per life stage 690 and predictor variable

Mixed linear model					
Life stage	Predictor variable	*F*-value	df	nom df	*P*-value
**Eggs**	Period	19.78	25	91	<0.0001
	Year	89.81	1	91	<0.0001
	Period*Year	10.29	22	91	<0.0001
**Crawlers**	Period	6.2	25	91	<0.0001
	Year	5.25	1	91	0.0243
	Period*Year	1.87	22	91	0.0209
**2nd Instar**	Period	4.52	25	91	<0.0001
	Year	0.32	1	91	0.5712
	Period*Year	3.39	22	91	<0.0001
**3rd/4th Instar**	Period	3.49	25	91	<0.0001
	Year	26.11	1	91	<0.0001
	Period*Year	2.51	22	91	0.0012
**Adult female**	Period	1.95	25	91	0.0117
	Year	1.88	1	91	0.1743
	Period*Year	5.88	22	91	<0.0001
**General linear model (ANOVA)**					
**Eggs**	Needle age class	40.93	2	423	<0.0001
**Crawlers**	Needle age class	56.72	2	423	<0.0001
**2nd Instar**	Needle age class	217.37	2	423	<0.0001
**3rd/4th Instar**	Needle age class	10.17	2	423	<0.0001
**Adult female**	Needle age class	173.64	2	423	<0.0001

**Fig. 4.**
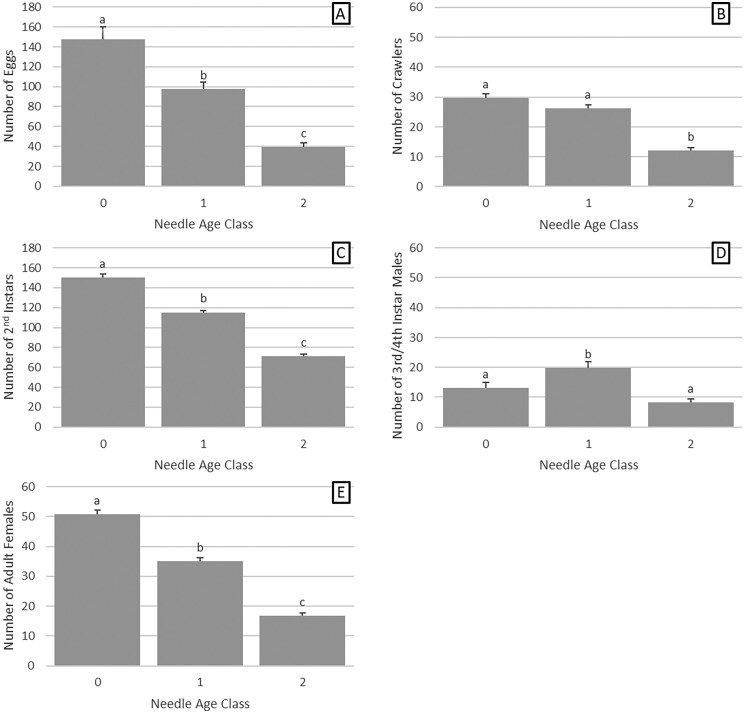
Average abundance and standard error of the mean of each of the 3 years’ branch segment across all locations sampled (239 trees at the UMRS, 233 trees at Mount Jefferson SP and 235 trees at the MRS) over the 2-year study period. The Needle Age classes are: 0 = new growth; 1 = growth from previous year; 2 = growth from two years prior. The distribution of abundance of the A) egg, B) crawler, C) 2nd instar, D) 3rd/4th instar male, and E) adult female stages. Bars with different letters for each life stage are significantly different at *P* ≤ 0.05 (Tukey-Kramer HSD test).

Needle age class significantly affected the abundance of all life stages (Fig. 4A–E). The numbers associated with needle age classes depict the age of the needles, where 0 is the most recent flush and 2 are needles produced 2 years ago. The mean abundance of all life stages were significantly lower on older needles than newly produced needles, except for the 3rd/4th instar male stage (Table 1). The abundance of 3rd/4th instar males is significantly higher on needles that are one year old (Fig. 4D).

## Discussion

Our results reveal that all elongate hemlock scale life stages are present on Fraser fir throughout the year, with multiple overlapping generations across the study sites in western North Carolina. This corroborates previous anecdotal observations on Fraser fir in North Carolina (Sidebottom and Bookwalter 2025) and studies conducted on hemlock (*Tsuga* spp.) outside our region (McClure 1978, Stimmel 1980, Abell and Van Driesche 2012). The lack of distinct peaks in abundance makes it challenging to evaluate the exact number of annual generations. Suggestions of two annual generations and a generation time as short as 16 weeks (Stimmel 1980, Abell and Van Driesche 2012, Sidebottom and Bookwalter 2025) can only be inferred from our results for egg abundance (Fig. 3A), but not from other findings. Egg abundance peaks twice in both sampling years, with peaks roughly 16 weeks apart.

The simultaneous presence of all life stages throughout the year has significant management implications. The crawler is the only stage not protected by armor, making it most susceptible to management actions (Stimmel 1980, Sidebottom and Bookwalter 2025). Pesticide application to manage this scale is thus generally conducted during peak crawler abundance (McClure 1978, Stimmel 1980, Sidebottom and Bookwalter 2025). While pesticide application during peak crawler emergence in the spring and summer may be the most effective approach to control elongate hemlock scale, it does not address crawlers that hatch after treatment providing residual populations for a subsequent reinfestation (McClure 1977a). In addition, pesticides are expensive, and overexposure could lead to resistance (Buss and Dale 2020).

Other components of IPM, such as biological control, can supplement pesticides (Sidebottom 2014). Several studies have identified and conducted efficacy experiments on known natural predators, parasitoids and entomopathogenic fungi to control elongate hemlock scale. This includes the parasitoid *Encarsia citrina* and fungi such as *Colletotrichum* spp., which have shown variable degrees of success controlling this scale in several studies across the eastern US (McClure 1977a, 1977c, 1979, Lambdin et al. 2005, Marcelino et al. 2009, Gouli et al. 2013). Although it effectively regulates elongate hemlock scale populations in its native range in Japan, *E. citrina* fails to control this scale reliably in its invaded range, due to the asynchronous lifecycles between parasitoid and host, likely brought about by the number of annual generations and presence of overlapping life stages (McClure 1986, Abell and Van Driesche 2012). One reason for the lack of consistent control of elongate hemlock scale by fungi such as *Conoideocrella* spp. are missing sexual structures on the host, suggesting the fungus requires an alternate host to reproduce (Urbina and Ahmed 2022). First observed by Urbina and Ahmed, *Conoideocrella luteorostrata* (Hypocreales: Clavicipitaceae) was conclusively isolated at UMRS, one of our sampling locations with ∼1% fungal infections, suggesting a high likelihood this was the fungus we observed during this study (Urbina and Ahmed 2022, Lovett et al. 2024). This corresponds with our results of low parasitization, suggesting that parasitoids and entomopathogenic fungi have little effect on scale populations in Fraser fir.

Our results suggest a significant drop in scale abundance following the 2nd instar. An anecdotal report suggests the natural elongate hemlock scale mortality to be over 30% (Sidebottom and Bookwalter 2025). However, the lack of parasitism fails to explain this substantial decline, suggesting other factors are responsible for the high mortality following the 2nd instar stage. Two possible factors are the length of the 2nd instar stage and competition. Little is known about the duration of individual life stages. However, both inter- and intraspecific competition for resources have been associated with slower development, reduced fecundity, and higher mortality in heavily infested hemlocks (McClure 1979). Once settled, often near the mother, crawlers become sessile, thus restricting their access to resources to what is within the reach of their stylet (McClure 1979, Greathead 1997). Increasing the duration of a life stage due to resource scarcity from competition increases the likelihood of observing and experience higher mortality of 2nd instars. This self-regulation subsequently decreases the number of adult females and 3rd/4th instar males that develop from them. Such density-dependent population dynamics have also been reported from hemlock woolly adelgid (*Adelges tsugae*), a relative of armored scale insects (Sussky and Elkinton 2014).

The relatively low proportion and abundance of crawlers compared to the 2nd instars can be partially explained by the greater mobility of crawlers (Figs 2 and 3B and C). Passive dispersal by wind occurs when an air current dislodges and moves the crawlers (Beardsley and Gonzalez 1975, McClure 1977b, Greathead 1997). Thus, it is likely that crawlers may be dislodged through other disruptive activities, such as sample collection, transportation to the lab, or microscopic analysis, partially explaining the lower abundance of crawlers observed.

Our observed average of 7 eggs per gravid female is similar to the 6 egg average reported on eastern hemlock (*Tsuga canadensis*) in Pennsylvania (Stimmel 1980), but lower than the fecundity reported on two Japanese and both eastern hemlock species in Connecticut (McClure 1983). In a common garden study conducted there, average fecundity on *T. sieboldii* and *T. diversifolia* were 20 and 18 eggs per female, respectively, and 12 and 16 eggs per female for *T. canadensis* and *T. caroliniana*, respectively (McClure 1983). The fecundity reported in Pennsylvania was lower due to only counting unhatched eggs. When accounting for hatched eggs by counting chorions, the actual fecundity was closer to 20 eggs per female. (Davidson and McComb 1958, Stimmel 1980). This is similar to our maximum count of 17 eggs and is consistent with anecdotal observations that suggest female elongate hemlock scale, on Fraser fir in NC, lay up to 16 eggs (Sidebottom and Bookwalter 2025). Our calculations are based on numbers obtained across all seasons and across three needle age classes, potentially negatively affecting the average number of eggs observed per gravid female. In addition, we did not account for empty chorions, potentially lowering our estimate of female fecundity.

In both years, peak egg abundance occurred between April and May and between August and October. The latter peak is of concern, because it is the largest and stretches into the Christmas tree harvest season, which typically starts in early November (Owen 2015, Dale et al. 2020). There are currently no ovicides to control elongate hemlock scale eggs, as they are protected under the cover of the mother. This results in scale hatching leading up to harvest and potentially as trees are being transported. There is concern that Fraser fir could act as media to disseminate this scale, as was demonstrated through agricultural inspections that detected the insect on Fraser fir Christmas trees and greenery as well as hemlock nursery stock in various states outside its current range (McClure and Fergione 1977, California Department of Food and Agriculture 2012, Stocks 2016, Oregon Department of Forestry 2019, Dale et al. 2020). Although no established populations have been observed, inspections in California, Florida, Oregon, and Wisconsin have found the scale on Fraser fir originating in North Carolina (California Department of Food and Agriculture 2012, Stocks 2016, Oregon Department of Forestry 2019, Wisconsin Department of Agriculture, Trade and Consumer Protection 2022). Shipping crawlers to new areas has the potential to start new populations, should they develop into an adult female (Dale et al. 2019). However, a suitable climate and hosts must be available. Research from Florida suggested that while several potential hosts allow initial feeding, they do not support development, while others supported up to three generations before further development failed (Dale et al. 2020).

Phenological variation is expected between locations and across seasons because aspects of population dynamics such as development and fecundity are affected by climatic and site conditions as well as host health and vigor (McClure 1978, Abell and Van Driesche 2012). With multiple overlapping generations per year, elongate hemlock scale exhibits a “flexible phenological life history pattern” where an insect may have lingering populations outside the window of its distinct generations (Salas-Araiza et al. 2008). The lack of distinct generations suggests that degree day models, which are generally used to predict insect development, are less precise with an insect such as this. Thus, understanding other factors that result in variation, such as weather, climate or location, can help anticipate peak abundance and more accurately drive management decisions. Regional differences in elongate hemlock scale phenology are well documented (McClure 1978, 1986, Abell and Van Driesche 2012). McClure (1978) found that climatic factors, such as frost-free period, significantly affected peak crawler emergence by several weeks between 2 locations in Connecticut just 15 mi (24 km) apart, documenting considerable variation in scale development over relatively short distances. The shortest aerial distance between sampling locations in our study was 7.17 mi (11.47 km) between UMRS and SP, which has statistically different average abundance of crawlers, 2nd instars, 3rd/4th instar males, and adult females. Over longer distances, phenology can differ by allowing for an additional generation per year, as is the case with this scale being univoltine or semivoltine in the northeastern U.S. and being bivoltine in the mid-Atlantic region (McClure 1978, Stimmel 1980, McClure 1986, Abell and Van Driesche 2012).

Despite significant variation over time and locations, the major takeaway is that all life stages are present at all locations throughout the year and that the duration and number of distinct cohorts or populations could not be determined with reasonable certainty. As is the case with other armored scales, the prepupal (3rd instar male) and pupal (4th instar male) stages are challenging to distinguish (Moran and Goolsby 2010). Adult male scales were not actively sampled, because they are tiny, winged insects with a relatively short life span and thus are difficult to intercept (Lambdin et al. 2005, Sidebottom and Bookwalter 2025). Intercepting adult male scale using sticky cards or utilizing emergence cages could give further indication of development duration, peak emergence, and mating patterns.

The fact that all elongate hemlock scale life stages can be found on the underside of Fraser fir needles of at least three age classes, has significant management implications. Fraser fir produces one annual flush per year (Owen 2015) and sheds needles after 5–7 years. Annual shearing is a common practice to give Fraser fir its iconic conical Christmas tree shape. It also can substantially increase canopy density (Owen 2009). Thus, older needles, 2 or more years old, are sheltered within the interior of the canopy, limiting the effectiveness of some application techniques, such as mist blowers, to achieve complete coverage (Yeary et al. 2018). This problem may be further exacerbated by field layout, where close spacing results in overlapping canopies as trees mature. Dense and overlapping canopies can provide refugia and facilitate pest movement (Yeary et al. 2018). Previous research on eastern hemlock in Connecticut suggests that scale resurgence occurs following incomplete pesticide application, due to the reproductive potential of residual populations and removal of beneficial organisms (McClure 1977a). These residual populations can have lasting implications on adjacent trees and fields or cause issues when trees are harvested and moved (Sidebottom 2016).

This study demonstrated that elongate hemlock scale produces multiple overlapping generations per year on Fraser fir in western North Carolina, resulting in the presence of all life stages throughout the year. We have observed variation in abundance across sampling locations, sampling periods, and sampling years, suggesting adaptations need to be made to accurately time scale management for specific locations and years. Notably, substantial peaks in egg abundance just prior to the harvest season are concerning and may necessitate additional management during this time to reduce crawler dispersal on cut Fraser fir trees and greenery. The presence of all life stages on needles up to 3 years old impacts scale management, as some pesticide applicators cannot provide thorough coverage on mature trees with dense canopies. The density dependent population dynamics implied by substantially lower abundance of post 2nd instars should be studied to inform the development of new management tools. Future research should focus on life stage abundance and development under controlled conditions to determine the impact of environmental factors on the presence and duration of individual life stages. Further, it could greatly benefit elongate hemlock scale management if future studies were to elucidate the exact nature of its reproduction and whether or not parthenogenic reproduction is a factor in the insect’s population dynamics. If a male is required, failure to mate by releasing pheromones has proven to successfully control spongy moth and could inhibit elongate hemlock scale reproduction and establishment in new areas.

## Supplementary Material

toag098_Supplementary_Data
