# Supplementary figures and images for: Phenology of the elongate hemlock scale (Hemiptera: Diaspididae) on Fraser fir Christmas trees in western North Carolina
Source: J Econ Entomol. 2026 Apr 23;119(3):2009–18. doi: 10.1093/jee/toag098 (PMC13268528; doi:10.1093/jee/toag098)

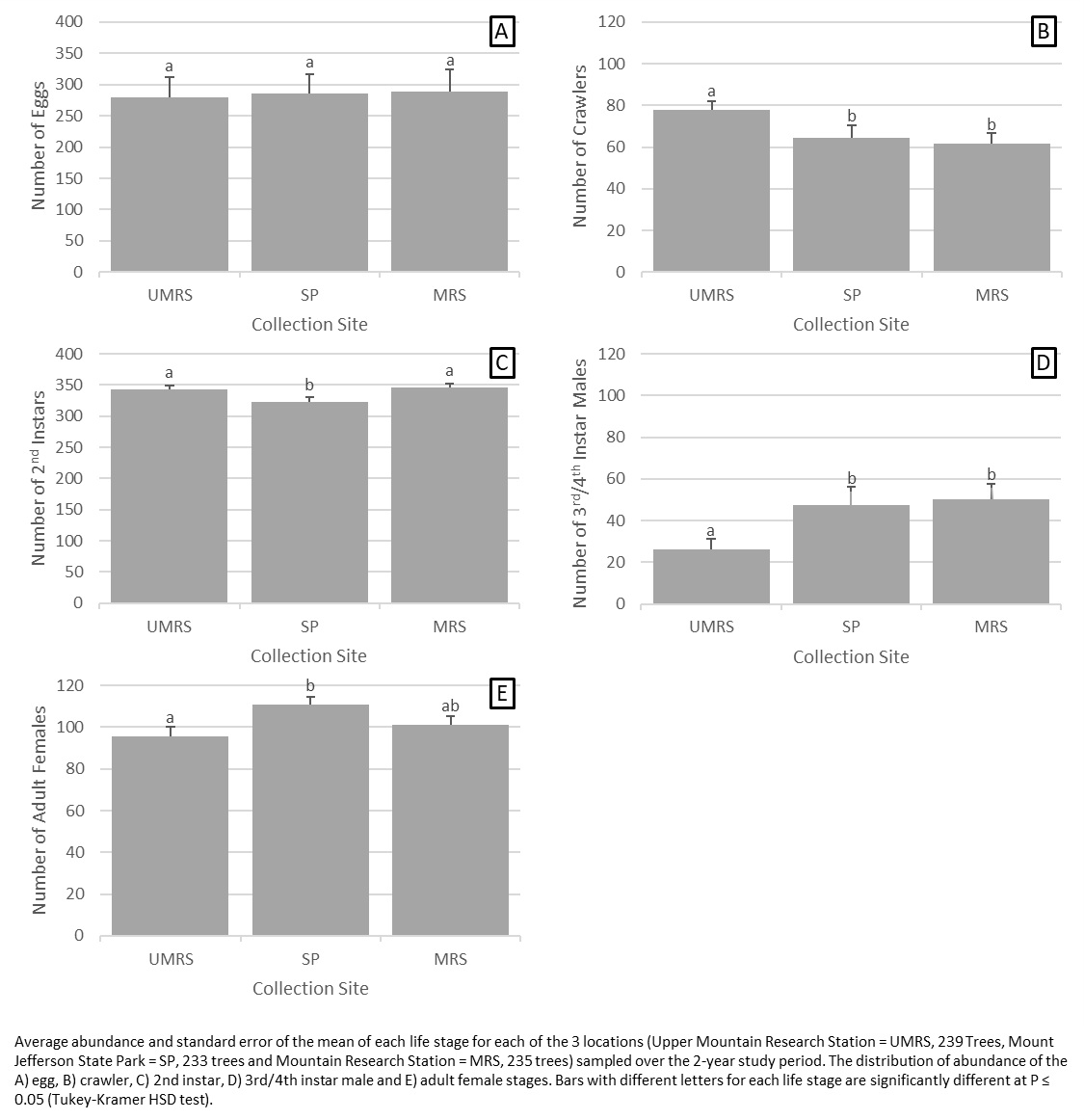

Supplement: toag098_Supplementary_Data [file toag098_supplementary_data.zip › toag098_Supplementary_Data.tif]
